# Digital Diabetes Health Coaching for Adults With Uncontrolled Type 2 Diabetes: Protocol for a Scoping Review

**DOI:** 10.2196/90739

**Published:** 2026-09-14

**Authors:** Fikadu Yehualashet, Adhanom Baraki, Karen Yeates, Catherine Harley, Kevin Woo

**Affiliations:** 1 School of Nursing, Faculty of Health Science, Queen's University, 92 Barrie Street Kingston, Kingston, ON, K7L 3N6, Canada, 1 613-533-2668; 2 School of Rehabilitation Therapy, Faculty of Health Science, Queen's University, Kingston, ON, Canada; 3 School of Medicine, Faculty of Health Science, Queen's University, Kingston, ON, Canada; 4 Nursing, Canadian Nurses Association’s, Nurses Specialized in Wound, Ostomy and Continence Canada (NSWOCC), Ottawa, ON, Canada

**Keywords:** AI, health coaching, type 2 diabetes, self-management, digital health, scoping review

## Abstract

**Background:**

Recent advancements in digital health have enabled the development of technology-enhanced diabetes coaching programs that offer personalized, data-driven, and adaptive support. However, evidence on their implementation and impact is scattered and has not been comprehensively synthesized. Although numerous studies have informed intervention development using different theories and models, the diversity of these approaches creates a significant challenge in developing an evidence-based intervention that is adaptable and effective across different population groups.

**Objective:**

This scoping review aims to systematically map and synthesize existing evidence on digital diabetes coaching and to identify critical gaps in the use of digital coaching among individuals with uncontrolled type 2 diabetes.

**Methods:**

This review will follow the Joanna Briggs Institute (JBI) methodology and the population-concept-context (PCC) framework to guide the search process. The review process will adhere to the PRISMA-ScR (Preferred Reporting Items for Systematic Reviews and Meta-Analyses Extension for Scoping Reviews) guidelines. A systematic search was conducted in PubMed, Embase, CINAHL, Cochrane Central Register of Controlled Trials, and APA PsycInfo. The methodological quality of the included studies will be appraised using JBI critical appraisal tools for randomized controlled trials (RCTs) and quasi-experimental studies, and the findings will be reported and contextualized descriptively.

**Results:**

This scoping review protocol was developed in September 2025 as part of a postdoctoral research fellowship at Queen’s University, funded through the Connected Minds program. The database search commenced in October 2025 and was completed in December 2025. The search strategy was finalized in consultation with a health sciences librarian, and a pilot-tested data extraction form was developed before full extraction. Findings from this scoping review are expected to be submitted for publication in 2026.

**Conclusions:**

This scoping review will provide a comprehensive and systematic map of existing evidence on digital diabetes coaching and will identify critical gaps in the literature. Evidence from this review will support the development of a digital diabetes coaching application for individuals with type 2 diabetes.

## Introduction

### Overview

Diabetes mellitus is a metabolic disorder related to impaired insulin secretion and/or insulin action, causing an imbalance in the hormones that regulate blood glucose [1-3]. Globally, an estimated 589 million adults aged 20 to 79 years were living with diabetes in 2024, accounting for 11.1% of the world’s adult population [4]. Based on projected trends in population growth, aging, and urbanization alone, the global diabetes burden is expected to increase by 45%, affecting an estimated 853 million people by 2050 [4]. Type 1, type 2, and gestational diabetes are the most common types [2], and type 2 diabetes is the leading cause of diabetes-related morbidity and mortality globally, accounting for 90% to 95% of all diabetes cases [3,5]. Diabetes mellitus has a significant impact both on affected individuals and on the broader community, contributing to over 80% of related medical expenditures and to reduced life expectancy and quality of life [3]. Diabetes mellitus adversely affects almost every organ of the human body, causing a wide range of acute complications, including diabetic ketoacidosis, hypoglycemia, and hyperglycemia, as well as serious chronic complications, including macroangiopathy leading to coronary artery disease, stroke, and hypertension, diabetic retinopathy causing vision loss and blindness, nephropathy resulting in renal insufficiency, and neuropathy manifesting as sensory disturbances and diabetic foot complications [6,7].

However, due to a lack of motivation; personal, financial, and social barriers; a lack of flexibility and autonomy associated with lifestyle changes; and health system–related problems, attaining and maintaining optimal health remains a challenge for the majority of individuals living with type 2 diabetes [8-10]. Ongoing diabetes self-management education and support are critical for empowering people, preventing acute and life-threatening complications, reducing the risk of long-term complications, and improving health-related quality of life [9-11].

Moreover, people require lifelong learning and ongoing support for self-management practices, sustained behavioral change, continuous support from family, health care providers, and peers, and regular clinical monitoring [10,12]. Diabetes self-management coaching has shown promising effects in enhancing self-management behaviors and improving glycemic control [13,14]. Health coaching is an emerging client-centered empowerment approach that shows promising effects for improving the clinical, behavioral, and psychological outcomes of people living with type 2 diabetes because it enhances diabetes self-efficacy, goal setting, and problem-solving skills [13,15,16].

The introduction of digital diabetes coaching presents an opportunity to address various social determinants that have historically hindered equitable access to effective diabetes care, education, and support for individuals with uncontrolled diabetes [12,17]. Further, AI has emerged as a transformative tool in health care, offering innovative solutions for prevention, diagnosis, disease management, and patient engagement [18-21]. Advancements in digital health technologies have led to the development of AI-driven self-management systems that support patient engagement and self-care [18,22]. AI has the potential to transform diabetes care by providing personalized, precise, and data-driven support to individuals living with diabetes and the health care professionals who serve them [19]. The integration of AI in diabetes care represents a paradigm shift from conventional management strategies to personalized and data-informed models of precision care [18,20]. AI-driven diabetes coaching uses machine learning algorithms, predictive analytics, and large language models, integrating patient data to deliver personalized, adaptive, and real-time guidance to enhance feedback loops [20,22,23]. These AI-driven health programs can collect and analyze real-time patient data and behavioral patterns to provide tailored recommendations, motivation, and problem-solving support that potentially enhance engagement in self-management and glycemic control beyond what is achieved with standard care [17,23].

However, the majority of the existing technology-based diabetes programs are focused on diagnosis, glucose monitoring, complication prediction, and insulin administration [24]. They rely on information provision, do not comprehensively address self-management elements, do not analyze and integrate patient self-care activity data, and typically lack linkage and interoperability with the health care system [25]. While several studies have investigated the effectiveness of digital or mobile application–based diabetes education and support programs, evidence on the effect of digital or AI-based diabetes self-management coaching programs is limited [24]. Despite growing interest in AI applications for diabetes care, there is no comprehensive review that has systematically examined and synthesized evidence on the effects of AI-based diabetes self-management coaching on clinical, behavioral, and psychosocial outcomes in individuals with diabetes.

### Literature Gaps

To address this evidence gap, a preliminary search was conducted for complete or ongoing studies on digital or AI-based diabetes self-management coaching in PubMed, PROSPERO (International Prospective Register of Systematic Reviews), and Open Science Framework (OSF) and for gray literature on Google Scholar and Google. No published or registered scoping or systematic review on digital or AI-based diabetes self-management coaching was identified. In the context of the rapidly rising global burden of diabetes, technology has emerged as a critical enabler of accessible, equitable, and client-centered diabetes care. Despite the advancement and expansion of AI-powered tools for the prediction and prevention of diabetes-related complications, evidence on the application of AI specifically for diabetes self-management remains limited, representing a critical gap that warrants further investigation [24]. In this regard, evidence shows that there is a lack of culturally adapted AI-based diabetes self-management coaching programs to support individuals with type 2 diabetes. Health coaching, as an emerging self-management support strategy demonstrating promising effectiveness in improving clinical and behavioral outcomes, is informed by diverse theories and models. This review will describe the theories, frameworks, and models underpinning digital diabetes health coaching interventions.

### Review Questions

This review will address the following three research questions: (1) What digital diabetes self-management coaching interventions have been developed and evaluated for individuals with uncontrolled type 2 diabetes? (2) What intervention components, delivery modalities, outcome measures, and study populations are represented in the literature? (3) What gaps exist in the evidence, particularly regarding AI-enabled, equity-informed, and culturally responsive coaching approaches?

## Methods

### Study Design

This scoping review will follow the Joanna Briggs Institute (JBI) evidence synthesis methodology to systematically identify relevant studies [26]. The reporting of the evidence will adhere to the PRISMA-ScR (Preferred Reporting Items for Systematic Reviews and Meta-Analyses Extension for Scoping Reviews) guidelines [27]. The protocol was registered in the OSF scoping review registry. This scoping review protocol was developed in accordance with the PRISMA for Protocols (PRISMA-P) 2015 statement [28], and the completed PRISMA-S checklist is provided as a supplementary file (Checklist 1).

### Search Terms and Keywords

The search strategy was guided by the population-concept-context (PCC) framework [29] and included the following key terms and related synonyms.

### Population

Participants in the studies included in the current review were adults with poorly controlled type 2 diabetes (HbA_1c_ ≥7%). Glycemic control is a gold-standard diagnostic and monitoring biomarker that indicates the accumulation of blood glucose in the previous 60 to 90 days [30]. The target glycemic level for the majority of individuals with diabetes is an HbA_1c_ level of less than 7%, while an HbA_1c_ level greater than or equal to 7% is considered an indicator of poor glycemic control. However, the target depends on the age of the individual with diabetes, duration of the illness, comorbidities, and history of severe complications, such as hypoglycemia [31]. Hence, the population of the current review comprises people with type 2 diabetes mellitus, and our search included related terms, such as “T2DM,” “type 2 diabetes,” “adult-onset diabetes,” “non–insulin-dependent diabetes,” “diabetes mellitus,” “poorly controlled type 2 diabetes,” and “uncontrolled diabetes.”

### Concept

The concept dimension of this review encompasses 4 interrelated elements: intervention components, theoretical frameworks, comparators, and outcomes.

Interventions of interest include digital diabetes self-management coaching programs delivered through any digital modality, such as mobile applications, web-based platforms, and other digital tools. Key terms related to these interventions include “health coaching,” “self-management coaching,” “diabetes coaching,” “self-management support,” “behavioral coaching,” “lifestyle coaching,” “digital health coaching,” “mobile coaching app,” “digital coaching,” “mHealth coaching,” “tele-coaching,” “remote coaching,” “behavior change intervention,” “motivational interviewing,” and “personalized self-management program.”

Theoretical frameworks refer to recognized health coaching theories, models, or frameworks that underpin the design and delivery of the intervention. Comparators of interest include standard care, didactic education, attention placebo control, and waitlist conditions. The primary outcome of interest in the reviewed studies is glycemic control, measured by HbA_1c_. Secondary outcomes include patient-reported outcomes such as diabetes self-care practices or activities, diabetes self-efficacy, diabetes-related distress, and health-related quality of life.

### Context

This review will consider the settings in which the included studies were conducted: health care facilities such as hospitals or primary care clinics, outpatient and ambulatory care settings, and community-based environments. Additionally, in terms of context, we will examine the types of technologies or delivery mechanisms used for diabetes self-management coaching, including online, web-based, or app-based platforms, mobile health (mHealth) and eHealth applications, digital health platforms, and telemedicine, telehealth, and remote monitoring systems that apply coaching as a behavioral change strategy.

### Screening of Eligible Studies

Two independent reviewers screened titles and abstracts, then assessed the eligibility of full-text articles. Disagreements between reviewers were resolved by discussion. The reviewers used Covidence software (Veritas Health Innovation Ltd) to facilitate screening for eligible studies, remove duplicates, assess risk of bias, and extract pertinent study characteristics and primary outcomes [32]. Studies were excluded from the review if they met any of the following conditions: the study population included participants younger than 18 years of age or individuals with type 1 diabetes, gestational diabetes, or mixed types of diabetes; coaching was not the primary intervention; the intervention was not delivered through digital or electronic platforms or mobile applications; the study design was not a randomized controlled trial (RCT), comparative observational study, or quasi-randomized study; the study was a review or study protocol; or there was no statement indicating that informed consent was obtained from participants.

### Eligibility Criteria

The eligibility criteria for the current scoping review included RCTs and comparative observational studies that assessed the effect of AI-based, digital, or technology-based diabetes coaching programs on clinical, behavioral, and psychological outcomes. Accordingly, the review will adhere to the inclusion and exclusion criteria outlined in Textbox 1.

Textbox 1. **Inclusion and exclusion criteria for studies on digital diabetes coaching.**

Inclusion criteria:

- Studies with participants who were adults with poorly controlled type 2 diabetes
- Studies with participants with uncontrolled blood glucose (HbA_1c_ ≥7%)
- Studies involving digital diabetes coaching programs delivered via mobile applications, web-based platforms, or any other technology
- Studies that used one or more theories, models, or frameworks of health coaching
- Studies that used standard care, didactic intervention, attention placebo control, or waitlist as comparison groups
- Studies conducted in health care facilities or community-based settings
- Studies that used online, web-based, or app-based platforms, mobile health (mHealth) and eHealth applications, digital health platforms, and telemedicine, telehealth, or remote monitoring systems and that applied coaching as a behavioral change strategy
- Published articles
- Studies published only in the English language

Exclusion criteria:

- Studies that included participants with type 1 diabetes
- Studies that included participants with gestational diabetes
- Studies that included participants with specific types of diabetes other than type 2 diabetes
- Studies that used only human coaching with no technological component
- Studies that relied on health education or knowledge acquisition and/or were based on diabetes education principles
- Studies that considered coaching that used only SMS- or text-based diabetes education
- Studies that were not conducted in health care facilities or community-based settings
- Conference proceedings

### Data Sources and Search Strategy

The search strategy was guided by the PRISMA-S guideline [33] and designed to address the research questions. A systematic literature search was run across the electronic databases PubMed, Embase, CINAHL, Cochrane Central Register of Controlled Trials, and APA PsycInfo and trial registries, including ClinicalTrials.gov, for publications dated through November 30, 2025. Reference lists of relevant studies, systematic reviews, and clinical guidelines were manually screened to identify additional eligible studies that may not have been captured through database searches. For studies whose full texts were not publicly available, corresponding authors were contacted by email to obtain the complete manuscript. In consultation with a Queen’s University librarian, a comprehensive search strategy was developed using predefined MeSH terms and relevant keywords. The search strategy used Boolean operators, using OR to combine synonymous and related terms within each concept and AND to intersect across the PCC domains, ensuring a search that was both sensitive enough to capture all relevant evidence and specific enough to exclude irrelevant literature. Accordingly, a search string was generated for the MEDLINE database and is provided in Multimedia Appendix 1.

### Filters and Limits

The following filters and limits were applied to the search strategy across all 5 databases. A publication date limit of January 2010 to November 30, 2025, was used to capture evidence coinciding with the emergence of digital and mobile health technologies in diabetes self-management. The search was restricted to English-language publications given resource constraints for translation. No study design or geographic filters were applied in keeping with the broad and inclusive scope of JBI scoping review methodology. Nonprimary research outputs, including editorials, commentaries, letters to the editor, and conference abstracts, were excluded from the search results.

### Data Extraction

The research team developed a data extraction form using an Excel spreadsheet to systematically extract relevant information from the included studies. To ensure quality and appropriateness, the data extraction tool was pilot-tested on 5 articles included in the review. Data extraction was conducted by 2 independent reviewers, and potential disagreements were resolved by discussion. The data extraction form captured the following elements: author(s); year of publication; country; study design; sample size; participant characteristics, including age, sex, and HbA_1c_ level; intervention details, including name of intervention, components, theoretical framework, delivery modality, platform type, duration, and frequency of use; comparators; primary and secondary outcomes measured, along with the measurement tools used; and key findings relevant to the review objectives (Multimedia Appendix 2).

### Risk of Bias and Quality Appraisal

Consistent with JBI and PRISMA-ScR guidelines, the methodological quality of the included studies was appraised descriptively to contextualize the evidence using JBI critical appraisal tools for RCTs and the JBI Checklist for Quasi-Experimental Studies, with findings reported narratively.

### Data Analysis

Data from the included studies will be presented using a standardized data extraction form addressing pertinent study characteristics and intervention features. The data synthesis will be structured to directly address the 3 guiding research questions of this review: (1) mapping the types of digital diabetes self-management coaching interventions developed and evaluated; (2) characterizing their components, delivery modalities, outcomes, and study populations; and (3) identifying gaps, particularly regarding AI-enabled, equity-informed, and culturally responsive approaches. The findings will be presented descriptively to summarize the included studies and scope of the evidence. Frequencies and proportions will be calculated and reported for key study characteristics including publication year, geographic region, country of origin, study design, sample size, population demographics, intervention type, digital delivery modality, theoretical framework, duration of intervention, comparator type, and outcomes assessed. These descriptive summaries will be presented in tables, figures, and narrative summaries to provide a clear overview of the existing evidence. Tables will be used to present data describing study characteristics and intervention features, while visual representations (eg, charts and evidence maps) may be used to illustrate the distribution of evidence across concepts and outcomes. The findings will be mapped to highlight the scope, diversity, and key characteristics of existing technology-based diabetes self-management coaching evidence.

### Dissemination Plan

Findings of this scoping review will be disseminated to academic, clinical, community, and policy audiences using multiple knowledge mobilization strategies. The full scoping review manuscript will be submitted to a peer-reviewed journal in 2026 to reach the scholarly community. Key findings will be presented at national and international conferences, including the Diabetes Canada Professional Conference and the International Medical Informatics Association World Congress. Plain-language summaries and infographics will be developed and shared through social media platforms, including LinkedIn and X, and through patient organizations and community health centers serving individuals with type 2 diabetes. The study findings will be used as foundational evidence for the development of an AI-based diabetes self-management coaching program and to identify key gaps to inform future research and clinical practice.

## Results

This scoping review protocol was developed in September 2025. A systematic search across 5 electronic databases was conducted between October and December 2025 in collaboration with a Queen’s University health sciences librarian. Over 2800 articles were imported into Covidence software for duplicate removal and initial screening. A standardized data extraction form was prepared and pilot-tested prior to full extraction. Two independent reviewers completed title and abstract screening, full-text review, and data extraction, and disagreements were resolved through discussion. The scoping review has been completed, and the manuscript is expected to be submitted to a peer-reviewed journal for publication in 2026. As this review draws exclusively from publicly available data sources (published articles) and does not collect primary data from human participants, ethical approval is not required. Findings of the scoping review will be disseminated through peer-reviewed journals, national and international conferences, and institutional and community knowledge-sharing events. Findings will also be integrated directly into the design and rationale of the AI-based diabetes coaching program, ensuring that the review generates not only scholarly output but real-world impact for individuals living with uncontrolled type 2 diabetes.

## Discussion

### Principal Findings

This scoping review is expected to generate a comprehensive and systematic evidence map of digital diabetes self-management coaching interventions for individuals with uncontrolled type 2 diabetes, addressing a critical and underexplored gap in the digital health literature. We anticipate identifying a heterogeneous landscape of digital coaching interventions that vary substantially in their components, delivery modalities, technological platforms, theoretical frameworks, and outcome measures. Based on the emerging literature, we expect that the majority of identified interventions will focus on glucose monitoring and information delivery, with limited integration of personalized, adaptive, and digital coaching features. The review is also expected to reveal inconsistencies in outcome measurement across studies, limited use of validated patient-reported outcome measures, and insufficient attention to implementation outcomes, such as feasibility, acceptability, fidelity, and equity of access. These anticipated findings will have direct implications for the design of future studies and clinical applications.

To our knowledge, no published scoping review has systematically mapped the evidence specifically on digital diabetes self-management coaching interventions for individuals with uncontrolled type 2 diabetes, distinguishing coaching from broader digital health or mobile health programs as a separate and theoretically grounded intervention approach. Prior systematic reviews and meta-analyses have examined the effectiveness of digital health technologies and mobile applications in diabetes management broadly, finding moderate improvements in glycemic control but noting significant heterogeneity in intervention design, population characteristics, intervention duration, and outcome measurement [34,35]. However, these reviews did not focus specifically on the coaching component, which is theoretically distinct from passive information delivery in its emphasis on self-efficacy, goal setting, problem-solving, and sustained behavioral change [36,37]. Existing reviews have also paid limited attention to digital diabetes coaching approaches, equity-informed design, and the implementation science dimensions of digital diabetes interventions [38]. This scoping review addresses these gaps directly by applying a systematic and inclusive methodology that captures the full breadth of digital coaching interventions, maps their key characteristics, and identifies where the evidence base remains underdeveloped, providing a foundation that prior reviews have not offered.

### Strengths and Limitations

This review has several important strengths. The application of rigorous JBI scoping review methodology, guided by the PCC framework and reported in accordance with PRISMA-ScR guidelines, ensures methodological transparency, reproducibility, and adherence to international standards for evidence synthesis. The comprehensive multidatabase search strategy, developed in consultation with a health sciences librarian and supplemented by a gray literature search, minimizes the risk of missing relevant evidence. The use of 2 independent reviewers for screening and Covidence software for study management further strengthens the rigor and reliability of the review process.

This review also has limitations that should be acknowledged. Searching was limited to articles published in English, which may introduce language bias and limit the representativeness of findings across non–English-speaking populations and health systems. The heterogeneity of digital coaching interventions in the literature may also make it challenging to draw definitive conclusions about effective intervention components or best practices. Additionally, the rapidly evolving nature of digital health and AI technologies means that new evidence may emerge during or after the review period that is not captured in our search.

### Future Directions

The findings of this scoping review will directly inform future research and practice on digital self-management of chronic health conditions. First, identified gaps in AI-enabled and equity-informed coaching approaches will provide a clear rationale and conceptual foundation for the development and evaluation of next-generation digital coaching platforms, including the AI-based diabetes coaching program currently under development by this team. Second, inconsistencies in outcome measurement identified across studies will support the case for developing a standardized core outcome set for digital diabetes coaching research, enabling more meaningful comparisons across future trials. Third, the limited evidence on implementation outcomes will highlight the need for future digital diabetes studies to adopt implementation science frameworks such as RE-AIM (Reach, Effectiveness, Adoption, Implementation, and Maintenance) and CFIR (Consolidated Framework for Implementation Research) to evaluate implementation process outcomes.

## Supplementary material

10.2196/90739 Multimedia Appendix 1 MEDLINE search string.

10.2196/90739 Multimedia Appendix 2 Data extraction tool.

10.2196/90739 Checklist 1 PRISMA-S checklist.

## Abbreviations

CFIR: Consolidated Framework for Implementation Research
JBI: Joanna Briggs Institute
mHealth: mobile health
OSF: Open Science Framework
PCC: population-concept-context
PRISMA: Preferred Reporting Items for Systematic Reviews and Meta-Analyses
PRISMA-ScR: Reporting Items for Systematic Reviews and Meta-Analyses Extension for Scoping Reviews
PROSPERO: International Prospective Register of Systematic Reviews
RCT: randomized controlled trial
RE-AIM: Reach, Effectiveness, Adoption, Implementation, and Maintenance
