# Supplementary material for: Digital Diabetes Health Coaching for Adults With Uncontrolled Type 2 Diabetes: Protocol for a Scoping Review
Source: JMIR Res Protoc. 2026 Sep 14;15:e90739. doi: 10.2196/90739 (PMC13682166; doi:10.2196/90739)
Supplement: Supplementary file 1 [file resprot-v15-e90739-s001.docx]

**Medline Search String**

| <https://proxy.queensu.ca/login?url=http://ovidsp.ovid.com?T=JS&NEWS=N&PAGE=main&SHAREDSEARCHID=5gwAoYGTrGdPRuaBjn8dv8iXles3A824x7ESPjgo4n9GknNtHMLKIwoSKA5LhtNCe> | | |
| --- | --- | --- |
| **#** | **Searches** | **Results** |
| 1 | exp Diabetes Mellitus, Type 2/ | 193941 |
| 2 | (non-insulin adj3 diabete*).ti,ab,kf. | 9118 |
| 3 | ((type II or type 2) adj3 diabete*).ab,ti,kf. | 217127 |
| 4 | (diabetes mellitus, type 2 or diabetes mellitus, stable or stable diabetes mellitus or diabetes mellitus, noninsulin dependent or diabetes mellitus, adult-onset or adult-onset diabetes mellitus or diabetes mellitus, adult onset or diabetes mellitus, ketosis-resistant or diabetes mellitus, ketosis resistant or ketosis-resistant diabetes mellitus or diabetes mellitus, non insulin dependent or diabetes mellitus, non-insulin-dependent or non-insulin-dependent diabetes mellitus or diabetes mellitus, type ii or niddm or diabetes mellitus, maturity-onset or diabetes mellitus, maturity onset or maturity-onset diabetes mellitus or maturity onset diabetes mellitus or mody or diabetes mellitus, slow-onset or diabetes mellitus, slow onset or slow-onset diabetes mellitus or noninsulin-dependent diabetes mellitus or noninsulin dependent diabetes mellitus or maturity-onset diabetes or diabetes, maturity-onset or maturity onset diabetes or type 2 diabetes or diabetes, type 2 or diabetes mellitus, noninsulin-dependent).ab,ti,kf. | 217927 |
| 5 | 1 or 2 or 3 or 4 | 278447 |
| 6 | exp Self Care/ or exp Self-Management/ or exp Self-Control/ | 81466 |
| 7 | (self adj3 (manag* or care or caring or regulat* or monitor* or efficacy)).ti,ab,kf. | 149858 |
| 8 | 6 or 7 | 200278 |
| 9 | 5 and 8 | 12207 |
| 10 | exp Mentoring/ | 5269 |
| 11 | (coach* or training or health counselling or motivational interviewing or mentor*).ti,ab,kf. | 729496 |
| 12 | 10 or 11 | 729900 |
| 13 | 9 and 12 | 1078 |
| 14 | (online or virtual or technology or telehealth or Phone or smartphone or video or audio or web or text message or mobile apps or iPhone or iPad or social media or WhatsApp or TikTok or remote* or AI or large language or artificial intelligence).ti,ab,kf. | 1616392 |
| 15 | 13 and 14 | 367 |
