# Supplementary material for: Digital Diabetes Health Coaching for Adults With Uncontrolled Type 2 Diabetes: Protocol for a Scoping Review
Source: JMIR Res Protoc. 2026 Sep 14;15:e90739. doi: 10.2196/90739 (PMC13682166; doi:10.2196/90739)
Supplement: Supplementary file 2 [file resprot-v15-e90739-s002.docx]

**Data Extraction Tool**

| Authors & Year | Country | Study Design | Population | Sample Size | Type of Intervention | Delivery modality | Description of Intervention | Platform type | Comparators | Duration of intervention | Frequency of use | Outcomes Assessed | Study setting | Key Findings | Theory / Model | Gaps / Limitation |
| --- | --- | --- | --- | --- | --- | --- | --- | --- | --- | --- | --- | --- | --- | --- | --- | --- |
|  |  |  |  |  |  |  |  |  |  |  |  |  |  |  |  |  |
|  |  |  |  |  |  |  |  |  |  |  |  |  |  |  |  |  |
|  |  |  |  |  |  |  |  |  |  |  |  |  |  |  |  |  |
|  |  |  |  |  |  |  |  |  |  |  |  |  |  |  |  |  |
|  |  |  |  |  |  |  |  |  |  |  |  |  |  |  |  |  |
|  |  |  |  |  |  |  |  |  |  |  |  |  |  |  |  |  |
|  |  |  |  |  |  |  |  |  |  |  |  |  |  |  |  |  |
|  |  |  |  |  |  |  |  |  |  |  |  |  |  |  |  |  |
|  |  |  |  |  |  |  |  |  |  |  |  |  |  |  |  |  |
|  |  |  |  |  |  |  |  |  |  |  |  |  |  |  |  |  |
|  |  |  |  |  |  |  |  |  |  |  |  |  |  |  |  |  |
